# Increased Osteocyte Lacunae Size and Organic Matrix Pyridinoline Content in Transiliac Bone from Patients with Axial Spondyloarthritis (axSpA)

**DOI:** 10.1007/s00223-025-01446-x

**Published:** 2025-12-01

**Authors:** Barbara M. Misof, Natalia P. Machado, Shibarjun Mandal, Markus A. Hartmann, Rafaela Ceron, Marcelo M. Pinheiro, Eleftherios P. Paschalis, Stéphane Blouin, Carolina A. Moreira

**Affiliations:** 1 Ludwig Boltzmann Institute of Osteology at the Hanusch Hospital of WGKK and AUVA Trauma Centre Meidling, 1st Med. Dept. Hanusch Hospital, Vienna, Austria; 2 Rheumatology Division, Universidade Federal do Paraná, Curitiba, Brazil; 3 Academic Research Center Pro Renal Institute, Curitiba, Brazil; 4 Rheumatology Division, Universidade Federal de São Paulo (UNIFESP/ EPM) - SP, São Paulo, Brazil; 5 Internal Medicine and Endocrine Division, Hospital de Clinicas da Universidade Federal do Paraná (SEMPR), Curitiba, Brazil

**Keywords:** Axial spondyloarthritis, Osteocyte lacunae, Bone matrix mineralization, Quantitative backscattered electron imaging, Pyridinoline, Raman microspectroscopy

## Abstract

Axial spondyloarthritis (axSpA) is a chronic inflammation disorder commonly associated with osteoporosis and fractures. For further information about the pathophysiology of the latter, we analyzed transiliac biopsy samples from n = 21 men with axSpA (age 46 ± 6 years, mean ± SD). This cohort comprised patients with (GROUP-1, n = 5) or without increased osteoid thickness and mineralization lag time by histology (GROUP-2, n = 16). We performed quantitative backscattered electron imaging for bone mineralization density distribution (BMDD) and osteocyte lacunae sections (OLS), and Raman microspectroscopy for bone material/compositional properties in cancellous (Cn.) and cortical (Ct.) bone compartments. Patients’ outcomes were compared to different respective reference (REF)/control (CTRL) data. The total cohort and subgroup GROUP-1 (considered separately) showed significant deviations in BMDD versus REF. The average degree of cancellous bone mineralization was decreased by − 1.8% (p < 0.05) in the total cohort and decreased by − 5.1% (p < 0.01) in GROUP-1, while GROUP-2 had normal BMDD. The total cohort and both subgroups showed abnormal osteocyte OLS-characteristics. Specifically, the respective increases in OLS-area in cancellous and cortical bone were + 14.4% and + 44.5% in GROUP-1 and + 8.8% and + 24.9% in GROUP-2. The total cohort as well as both subgroups showed additionally deviations in organic matrix properties from CTRL with an increased pyridinoline content versus CTRL in both the cohort and subgroups. Our findings showed abnormal bone matrix mineralization in these patients with axSpA, with the largest deviations in the subgroup with histologically defined mineralization defects. The also observed altered osteocyte morphology and pyridinoline content might suggest osteocyte and osteoblast functional abnormalities in axSpA.

**Supplementary Information:**

The online version contains supplementary material available at 10.1007/s00223-025-01446-x.

## Introduction

Axial spondyloarthritis (axSpA) is a male pre-dominant, human antigen (HLA)-B27 associated inflammatory disease of the spine [1]. Typically, it begins with sacroilitis and evolves over years to include ascending spinal involvement and calcifications of ligaments [2]. Patients suffer from back pain, stiffness, and limited motion of the lumbar and thoracic spine. They are usually younger than 45 years at the onset of these symptoms [3]. Clinical diagnosis is complex and typically comprises either the presence of radiographic sacroilitis or in its absence, sacroilitis on magnetic resonance imaging and positivity for HLA-B27 together with clinical criteria [3]. HLA-B27 is a genetic marker for axSpA found in most patients [1]. There is ongoing search for bone markers, which are helpful at early stages of the disease, before radiographic evidence of structural changes is visible, as summarized in review articles [4, 5]. In any case, low bone mineral density (BMD) together with increased prevalence to fragility fractures was reported to be a significant comorbidity in patients with axSpA [6–8]. Despite the large variation of reported data on prevalence in the literature, there is clear evidence for a general bone involvement and systemic bone loss in this disorder [6–9]. In their review, van der Weijden and colleagues showed that even patients with less than ten years of disease duration have a high risk of decreased bone mass and osteoporosis (prevalence of 51–54% and 13–16%, respectively) [10].

This “paradox” of osteopenia and osteoporosis together with increased new bone formation (syndesmophytes and bone spur), which might lead to ankyloses, however remains to be elucidated. Osteopenia might be related to the chronic local inflammation and/or systemic factors, which inhibit bone formation and/or stimulate bone resorption, but also partly to decreased mobility of patients in late disease stages.

For more information about the pathophysiology of the bone involvement in axSpA, studies characterizing the bone tissue/material alterations in this disorder are needed. However, studies of human bone material and likewise from animal models of axSpA are sparse. HLA‐B27 transgenic rats show many clinical features similar to human axSpA, like abnormal low trabecular bone density, decreased mechanical quality of their bones and altered bone material characteristics, including enhanced advanced glycation end products content [11]. Transiliac bone biopsy studies of patients evaluating bone histomorphometry indicated lower rather than higher bone turnover in axSpA compared to healthy reference population [2, 12–14]. Part of the current patient cohort with axSpA presented with abnormally thick osteoid seams and increased mineralization lag time [12].

Given the aforementioned high skeletal fragility reported for patients with axSpA [6], we hypothesized that also alterations in bone material/tissue might occur in this disorder. For this purpose, we studied the bone matrix, in particular the mineral content and its distribution, the morphology of the osteocyte lacunae (i.e. the osteocyte lacunae sections within the mineralized bone matrix) and properties of the mineral and organic matrix in biopsy samples from a previously published cohort of male patients with axSpA [12]. Mineral content is a characteristic of bone material and independent of bone volume, thus, providing additional information to the well-known bone mineral density by dual X-ray absorptiometry (BMD by DXA) that is known to be low in patients with axSpA. Knowledge of mineral content, properties of the organic matrix, as well as characteristics of osteocyte lacunae might contribute to the further understanding of the systemic skeletal abnormalities in axSpA.

## Materials & Methods

### Patients

We have studied transiliac bone biopsy samples from n = 21 male patients with the diagnosis of radiographic axial spondyloarthritis (axSpA). The study was approved by the Ethics Committee of Federal University of Parana (Protocol no. 94131618.3.0000.0096). These patients have been described previously for clinical characteristics, including disease history, medication, serum data, as well as DXA measurements [12]. For demographic and important clinical characteristics see Table 1. The patients’ biopsy samples have been analyzed for static and dynamic histomorphometry [12]. For the latter, all patients received tetracycline hydrochloride (500 mg, twice a day, orally) in the following labelling schedule: 3 days ON tetracycline hydrochloride, followed by a 10-day interval OFF the medication, and an additional 3 days ON tetracycline hydrochloride. The transiliac biopsy was performed 3–5 days after the last day of tetracycline administration [12]. The previous histomorphometric evaluation showed that part of the patients presented with osteoid thickness larger than 12.5 μm and mineralization lag time longer than 100 days [12], i.e. these patients met criteria for osteomalacia as given in [15]. However, the bone samples of these patients also showed clearly defined tetracycline labels, therefore, we denote this group of patients having signs of histologic “mineralization defects” (n = 5, GROUP-1), while the remaining cohort presents without histologically defined mineralization defects (n = 16, GROUP-2). These two groups were considered separately for bone matrix mineralization and osteocyte lacunae sections characteristics as well as Raman microspectroscopic outcomes additionally to the total cohort when compared to respective reference/control data.

**Table 1:** Demographic and clinical characteristics of the cohort of male patients with axSpA

	Patients with AS (*n* = *21*)
Age (yrs)	47 [42; 49]
Disease duration (yrs)	16 [9; 28]
Disease activity	Inactive n = 4 Low n = 2 High n = 10 Very high n = 5
Medication	
Current NSAID use (nb. of patients)	12
Synthetic or biologic DMARDs (nb. of patients)	9
Densitometric classification according to WHO criteria of osteopenia/osteoporosis^1^	
T-score or Z-score ≥ -1.0	11
-2.5 < T-score or Z-score < -1.0	7
T-score or Z-score ≤ -2.5	3
positive for HLA-B27 (nb. of patients)	19

^1^Meeting the criteria at the lumbar spine and/or femoral neck

### Bone Biopsy Samples

The block samples, which were previously used for histomorphormetric characterization, were ground, polished (PM5 Logitech, Logitech, Glasgow, UK) for Raman microscpectroscopy and subsequently coated with a thin layer of carbon (Agar SEM Carbon Coater, Agar, Stansted, UK) ensuring electrical conductivity for consequent imaging in the scanning electron microscope [16, 17].

### Quantitative Backscattered Electron Imaging (qBEI)

For the quantification of the mineralization densities in the bone biopsy samples, digital images of the samples were collected using a digital scanning electron microscope (SEM, Supra 40, Zeiss, Oberkochen, Germany) equipped with a four-quadrant semiconductor backscattered electron (BE) detector. For the acquisition of 8-bit grey scale images, the following setting of the SEM was used: 10 mm sample-detector-distance, 20 kV acceleration voltage, probe current between 280 and 320 pA, and 1.8 μm spatial (pixel) resolution. Calibration of the grey levels was performed as described previously using high purity aluminum/carbon reference samples [16]. The pixel grey levels in calibrated qBEI images can be transferred to local calcium concentrations (the brighter the pixel of the image the higher the local calcium concentration). Noteworthy, osteoid is commonly not visible in the calibrated qBEI images.

#### Bone Mineralization Density Distribution (BMDD)

**Fig. 1:**
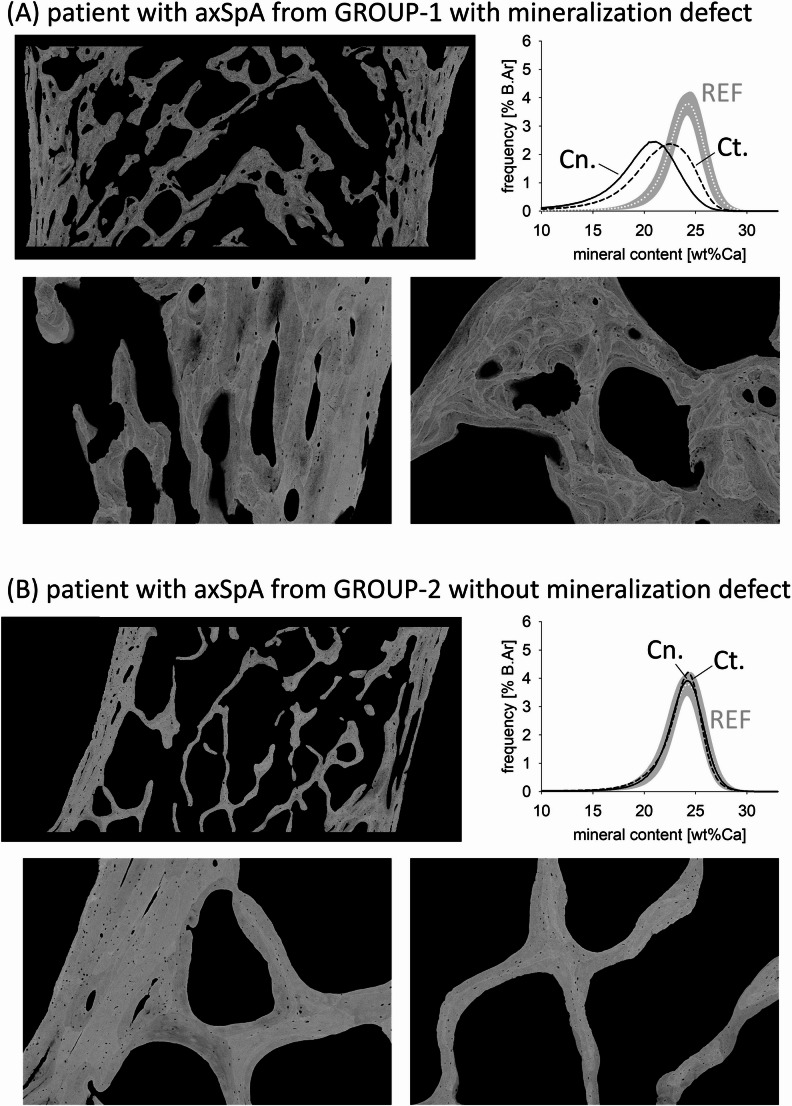
qBEI overview and detail images of one example of transiliac bone biopsy and corresponding BMDD from each studied subgroup. **A** Cross-sectional area of the transiliac biopsy sample from a patient with a mineralization defect (GROUP-1). The patient’s previously measured O.Th was 18.21 μm and Mlt was 104 days (therefore, the patient was histologically classified to present with mineralization defects [12]). The volume of mineralized bone matrix (md.BV/TV based on qBEI image) of the patient was 34%, which is relatively high compared to the other biopsy samples (however, it is lower than the histomorphometric BV/TV of 48%, which is including also the large amount of osteoid in this samples, which the latter is not visible in qBEI images). Clearly visible are the abnormally small bone packets together with large areas with low degree of mineralization. In part, the bone packets are delineated by dark (i.e. lowly mineralized) instead of normally bright (highly mineralized) cement lines. Furthermore, the osteocyte lacunae appear larger than normal. **B** qBEI images of an example of a transiliac bone biopsy from a patient without mineralization defects. The patients’ bone volume and Ct.Th are lower than those seen in (**A**). The patient’s BMDD is within the normal range

The qBEI images can be analyzed for the frequency histogram of calcium concentrations that are present in the bone sample by calculation of the bone mineralization density distribution (BMDD). The BMDD measures the frequency of a certain calcium concentration (calcium weight %, Ca wt.%) in percent of mineralized bone area (%B.Ar) (Fig. 1). For each patient, the BMDD was obtained from the entire sectioned biopsy surface, for cancellous (Cn.BMDD) and cortical (Ct.BMDD) compartments separately. Five parameters were derived from the BMDD as defined previously [17]: CaMean = the mean calcium content, CaPeak = the most frequent calcium content (i.e. the peak position of the BMDD curve), CaWidth = the full width at half maximum of the BMDD (a measure for the heterogeneity of mineralization), CaLow and CaHigh = percentages of lowly and highly mineralized bone areas (with respect to the 5th and 95th percentile of a reference population).

**Fig. 2:**
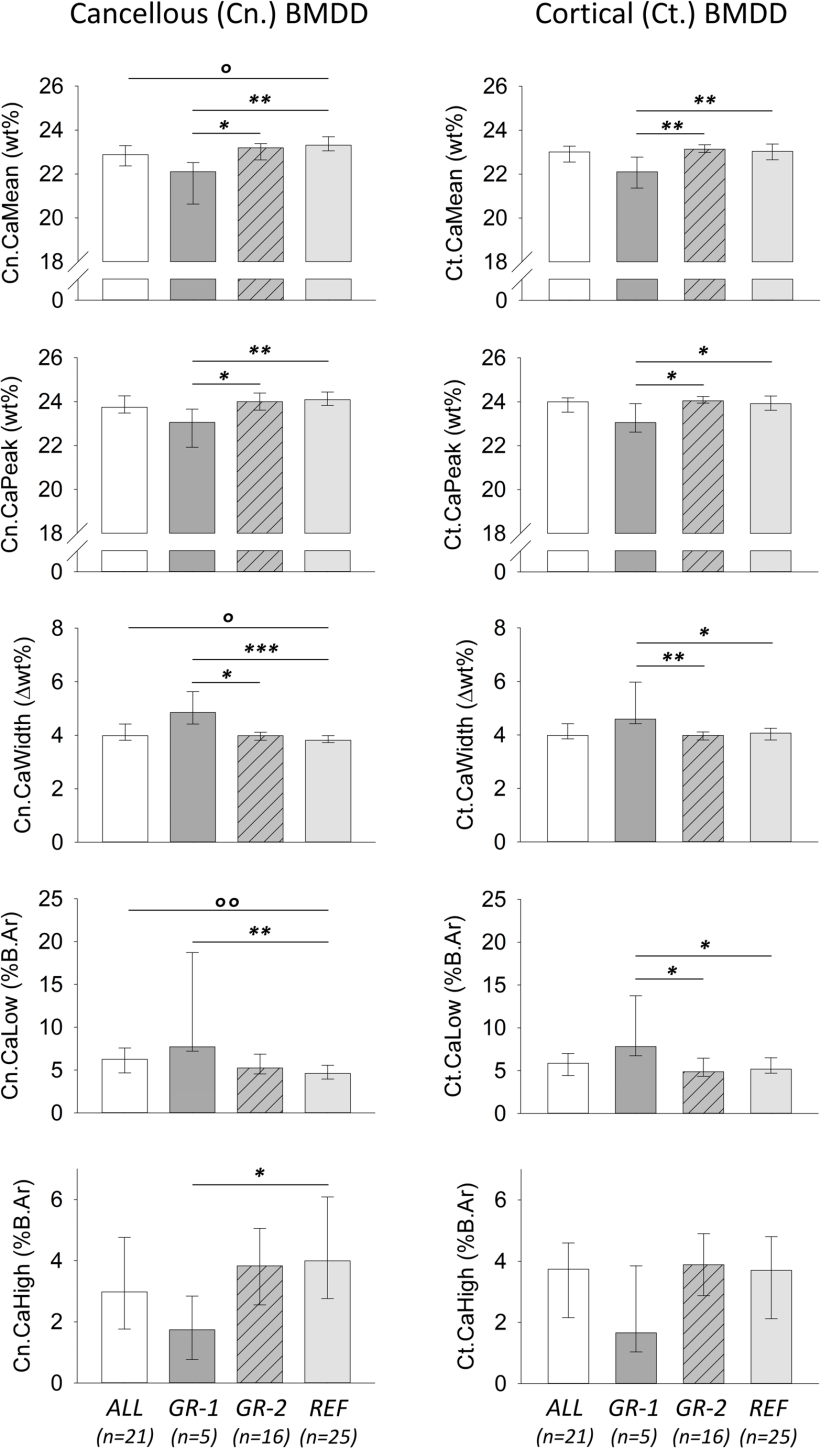
Bar chart with the BMDD outcomes from patients with axSpA (ALL indicates total group, GR-1 and GR-2 indicate GROUP-1, n = 5, and GROUP-2, n = 16, respectively) and reference BMDD from adult healthy individuals (REF, n = 25) published previously [16]. Data show median and IQR, significant p-values shown (°p < 0.01, °°p < 0.001) are either based on t-test or Mann–Whitney rank sum test comparison between the entire group with REF or are results from posthoc pairwise comparison (*p < 0.05, **p < 0.01, ***p < 0.001) following significant ANOVA or ANOVA on ranks comparison. Cancellous BMDD-outcomes are shown at left, cortical outcomes at right. The decreases in average (CaMean) and typical (CaPeak) calcium concentrations together with the increases in mineralization heterogeneity (CaWidth) and percentage of lowly mineralized areas (CaLow) reflect a general shift of the BMDD to lower calcium concentrations (see total cohort and GROUP-1). In contrast, no significant deviation in BMDD outcomes from references is seen in GROUP2

The BMDD outcomes of this study were compared to published general reference data from transiliac bone biopsy from healthy adults [16]. Median and interquartile range of this reference are shown in bar charts in Fig. 2. These reference data were obtained from autopsy iliac crest bone samples from n = 25 healthy adult individuals (7 males, 18 females, mean ± SD of age = 68.7 ± 19.0 years, age range = 37 to 95 years).

#### Osteocyte Lacunae Sections (OLS) Characteristics

For OLS-analysis, qBEI images with 0.88 µm/pixel spatial resolution were collected from both the cancellous and cortical compartments. Based on a custom-made macro in ImageJ software (version 1.52; NI, Bethesda, MD, USA) the lacunae were separated from surrounding mineralized bone matrix (calcium threshold of 5.2 wt.%Ca), resulting in binary images showing the OLS void area and the mineralized bone tissue. Furthermore, a size threshold between 5 μm^2^ and 200 μm^2^ was employed to distinguish OLS from larger channels (like vascular pores or cracks), as described previously [18]. The binary images were analyzed for 5 parameters characterizing the OLS in cancellous and cortical compartments separately: OLS-density (OLS-number/mm^2^) = the number of OLS per (mineralized bone matrix area + OLS total area);

OLS-porosity (%) = OLS total area per (mineralized bone matrix area + OLS total area);

OLS-area (µm^2^) = median value of the OLS areas per each sample;

OLS-perimeter (µm) = median value of the OLS perimeters per each sample;

and OLS-aspect ratio = median value of the OLS-aspect ratios per each sample. The OLS-aspect ratio is a measure for the shape of the OLS, which is defined as the ratio of the long over the short half-axis of a fitted ellipse to the section. A value of 1 indicates a perfect circle, while increasing values indicate an increasing elongated shape. OLS with aspect ratio values > 10 were excluded from the analysis.

**Fig. 3:**
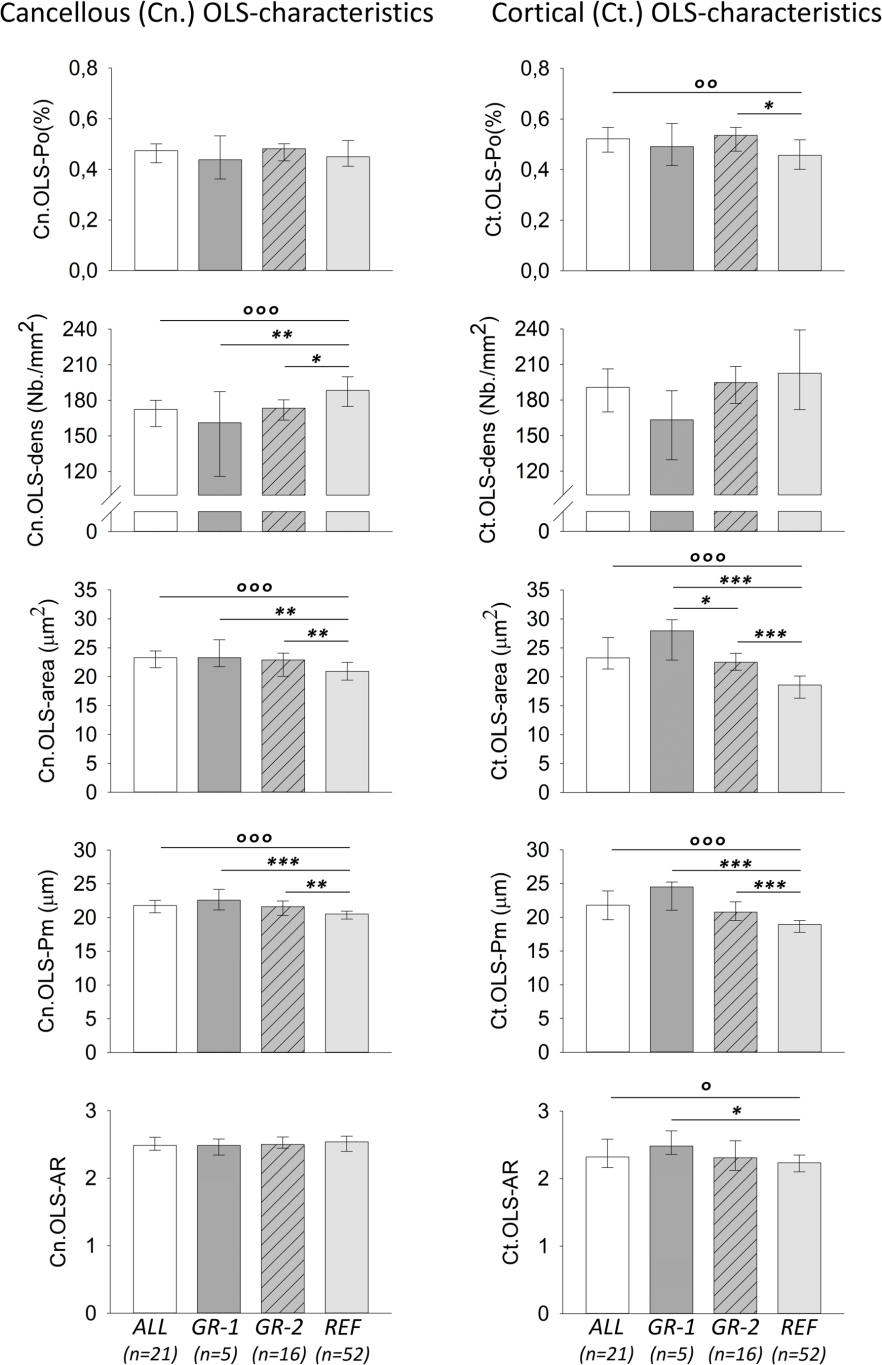
Results of OLS-characteristics from the total cohort (ALL, n = 21), GROUP-1 (GR-1, n = 5), and GROUP-2 (GR-2, n = 16) in comparison to reference (REF, n = 52) data published previously [18]. Median and IQR are shown from cancellous (left) and cortical (right) compartments. °p < 0.05, °°p < 0.01, °°°p < 0.001 indicates the results of the t-test or Mann–Whitney rank sum tests (ALL versus REF), while *p < 0.05, **p < 0.01, ***p < 0.001 shows significant difference according to pairwise post-hoc tests following ANOVA or ANOVA on ranks (comparing GROUP-1, GROUP-2 and REF)

The obtained OLS-outcomes of bone from the patients were compared to published reference data from iliac crest bone samples from healthy adults [18]. This OLS-reference group comprised adult women older than 30 years (age mean ± SD = 50.6 ± 17.7 years, range 31–95 years). The median and interquartile range of these OLS-reference data are shown in the bar charts in Fig. 3.

### Raman Microspectroscopy

Raman microspectroscopy was performed using a Senterra Raman microscope (Bruker Optik GmbH, Ettlingen, Germany) with a continuous laser beam with a wavelength of 785 nm and a power of 100 mW. The laser was focused onto the sample through a 50 × objective on a Raman fluorescence microscope (Olympus BX51, Hamburg, Germany), providing a spatial resolution of about 0.6 µm. The block samples were placed on a motorized XY stage (Bruker Optik GmbH) for analysis. Raman measurements were taken at specific microanatomical regions, particularly actively forming surfaces in cortical (osteons) and cancellous bone, referred to as regions of interest (ROIs), identified using the fluorescent tetracycline labels (Fig. 5a, b).

Within each ROI, the specific microanatomical locations examined included the osteoid-forming front (Os) (1 μm from the mineralization front, Fig. 5a, b), the area between the second label and the mineralizing front (TA1), the region between both fluorescent labels (TA2), and the area behind the first label (TA3). These areas are considered to provide information about the kinetics of the early phase of bone mineralization/material maturation. Each biopsy provided six ROIs comprising three from actively forming osteons in cortical bone and three from the actively forming cancellous bone regions. ROIs with only a single label or overlapping labels were excluded from analysis. For analysis, values from equivalent anatomical regions were averaged across ROIs, specific to cortical or cancellous bone, and this average value was treated as a single statistical unit. Selected interstitial bone regions considered at a distance of typically 20–35 μm from surfaces with evident fluorescent labels were also measured, indicative of older tissue age (marked in yellow in Fig. 5a, b) thus providing information about later secondary mineralization/material maturation.

Raman spectra were acquired using a thermoelectrically cooled charge-coupled device (CCD; Bruker Optik GmbH) and processed with the OPUS 6.5 software package (Bruker Optik GmbH). Spectra were trimmed to the 350–1800 cm⁻^1^ range and corrected for fluorescence background using the rubber band method with five iterations. No further spectra manipulation was performed. If cosmic spikes were evident in any measured spectra, they were remeasured. The following Raman parameters were calculated, as published elsewhere [19]: (i) Mineral/Matrix Ratio (MM): MM calculated from the integrated areas of the v_2_PO_4_ band (410–460 cm⁻^1^) and the amide III band (1215–1300 cm⁻^1^). This ratio considers the quantity of mineral and organic matrix present in the analyzed microvolume and is independent of tissue organization and orientation [20]. (ii) Glycosaminoglycan (GAG) Content: calculated by the GAG/matrix ratio, which is the ratio of the integrated areas of the proteoglycan/CH₃ band (1365–1390 cm⁻^1^), indicative of mucopolysaccharides, to the Amide III band (1215–1300 cm⁻^1^). (iii) Nanoporosity: The sub-micron porosity is estimated by the ratio of the integrated areas of the spectral slice from 494 to 509 cm⁻^1^ (representing the embedding material) to the Amide III band. (iv) Pyridinoline (Pyd) Content: The Pyd, which is an enzymatic trivalent collagen cross-link, is calculated using the absorbance height at 1660 cm⁻^1^ divided by the area of the amide I band (1620–1700 cm⁻^1^). (v) Mineral Maturity/Crystallinity (MMC): approximated by the inverse of the full width at half height (FWHH) of the v_1_PO_4_ band (930–980 cm⁻^1^).

To streamline the calculation, a custom Python (https://www.python.org/) script was developed using the pandas (https://pandas.pydata.org/), numpy (https://numpy.org), scipy (https://scipy.org), and brukeropus (https://github.com/joshduran/brukeropus) libraries. For each spectrum, the script calculated the area under the curve within specified wavenumber ranges using the trapezoidal rule (scipy.integrate.trapezoid) and extracted intensity values at defined wavenumber (1660 cm^−1^).

During Raman measurement, the operator remained blinded until all raw spectroscopic data were collected. Patients’ outcomes were compared to data measured in iliac crest autopsy samples from a healthy control group (CTRL), which comprised n = 7 men aged 61.9 ± 15.7 years (mean ± SD).

### Statistical Analysis

Data obtained from qBEI analysis: We used SigmaStat for Windows Version 4.0 (SPSS Inc.) for statistical analysis of BMDD- and OLS-parameters. First, we compared the outcomes from the total study group with the REF data by t-test or Mann–Whitney rank sum test (depending on passing or failing of Brown-Forsythe equal variance and Shapiro–Wilk normality tests, respectively). Second, the two subgroups of the axSpA cohort GROUP-1 and GROUP-2 (with or without mineralization defects as defined by previous histomorphometric analysis [12], respectively) were compared to normative data (BMDD-REF or OLS-REF accordingly). We used ANOVA or non-parametric Kruskal–Wallis ANOVA on ranks (depending on passing or failing of Brown-Forsythe equal variance and Shapiro–Wilk normality tests, respectively). These tests were used as TwoWay repeated measures ANOVA (with the factors group) and site (cancellous, cortical) showed a significant interaction for most parameters. The comparison of OLS-characteristics among the patients of GROUP-2 according to their type of treatment (NSAIDs versus immunobiologic treatments) was performed using t-tests or Mann–Whitney rank sum tests (depending on passing or failing of equal variance and normality tests, respectively).

Additionally, correlation analysis of Cn. BMDD versus Ct.BMDD as well as BMDD and OLS outcomes with disease related clinical parameters (duration and activity of the disease), as well as serum phosphorus, PTH, vitamin D level, and with histomorphometric dynamic indices of bone formation (MS/BS, MAR, BFR/BS) was performed based on Pearson Product Moment (PPM) correlation.

Data from Raman microspectroscopy: Outcomes of the total cohort versus CTRL or the two subgroups versus CTRL were compared by 2way ANOVA, with anatomical position / tissue age, and patient group as the two factors.

Two-tailed p < 0.05 was considered significant.

## Results

### BMDD from Patients with axSpA

**Fig. 4:**
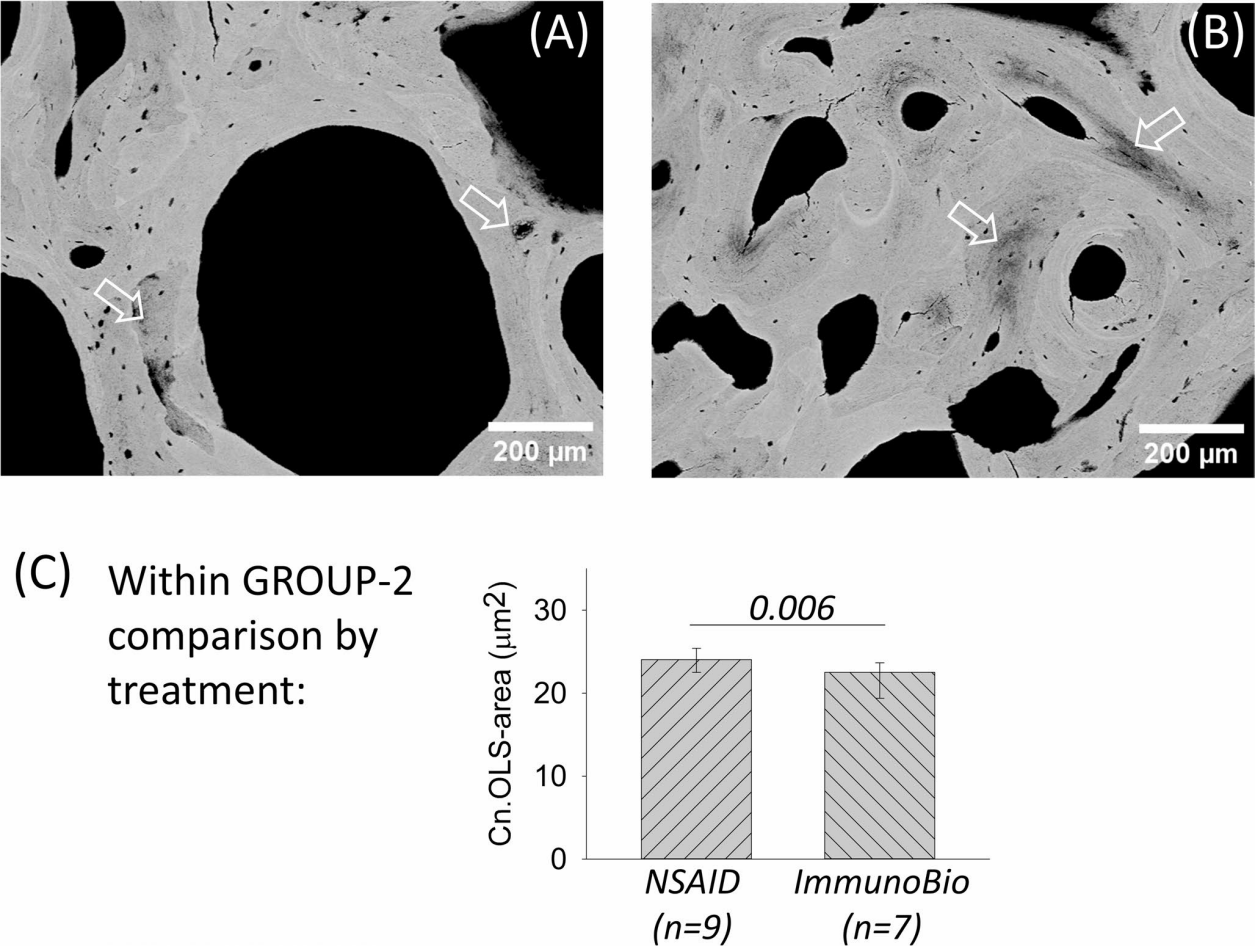
qBEI observations within GROUP-2. **A** and **B** show detail images of cortical bone from two patients. These images give evidence for small bone areas with abnormal mineral content (see arrows) within the otherwise normally mineralized bone matrix. Furthermore, in **A** also an osteocyte lacunae with an abnormally lowly mineralized perilacunar bone area is shown (right). **C** Within GROUP-2, patients with NSAID treatment had significantly larger OLS-area than those with immunobiologic treatment. Bars represent median and interquartile range, p-value based on t-test

Typical qBEI images as well as BMDD curves of examples of each GROUP-1 and GROUP-2 are shown in Fig. 1A and B, respectively. GROUP-1 comprised one transiliac bone biopsy sample with clear evidence of mineralization defects (shown in Fig. 1A). This biopsy sample presents with an abnormally high heterogeneity of mineralization densities (CaWidth) including extremely low mineral content of the bone matrix at the bone surface and/or beneath the surface (in particular at cement lines) and in the surrounding bone area to the osteocyte lacunae (Fig. 1A). The other biopsy samples of GROUP-1 also showed some of the aforementioned signs of mineralization defects, however to a lower extent. The biopsy samples of GROUP-2 presented with a lower variety in mineralization densities, i.e. lower CaWidth. There was no evidence of larger areas of mineralization defects in this patient group. However, smaller focal mineralization defects could be observed also in GROUP-2. In Fig. 4A and B, we show two examples of cortical bone from GROUP-2, which comprise small areas of very low mineralization within the otherwise normally mineralized bone matrix. In one image, also the hypomineralized area around an osteocyte lacunae can be seen. As these areas of abnormally low mineral content are limited and occur in few of the samples of GROUP-2, they did not significantly alter the overall BMDD outcomes in this patient subgroup when compared to reference data (REF) (see below).

In Suppl. Table 1 and Fig. 2, the outcomes of the BMDD-parameters and their comparison to REF are summarized. When considering the entire study cohort, significant difference to REF could be observed in cancellous bone only (by t-test or Mann–Whitney rank sum test). Cn.CaMean was decreased while Cn.CaWidth and Cn.CaLow were increased compared to REF. When comparing the two subgroups GROUP-1 and GROUP-2 at once with REF by ANOVA or ANOVA on ranks, we found that all Cn.BMDD- and Ct.BMDD-parameters (except for Ct.CaHigh) differed significantly between these three groups. GROUP-2 neither differed in cancellous nor in cortical BMDD from BMDD-REF. In contrast, GROUP-1 showed a significant shift to lower mineralization densities compared to BMDD-REF, comprising decreased cancellous and cortical CaMean, CaPeak and increased CaWidth and CaLow. CaHigh was decreased in cancellous bone but not significantly different in the cortex (p-values of these comparisons ranging from < 0.05 to < 0.001, for details see Suppl. Table 1). Additionally, several BMDD-parameters from GROUP-1 were significantly different to those of GROUP-2 (see Suppl. Table 1 and Fig. 2).

Cancellous BMDD parameters showed highly significant positive relationships with the corresponding cortical BMDD parameters. For instance, Cn.CaMean was positively correlated with Ct.CaMean (PPM correlation coefficient R = 0.91, p < 0.0001). The PPM correlation coefficients R of these correlations between cancellous and cortical BMDD-parameters were ranging from 0.74 to 0.93 (all p ≤ 0.0001).

We did not observe a significant relationship of the BMDD outcomes with previously measured clinical parameters of disease duration and activity score or with serum phosphorus, PTH or vitamin D levels (with the exception of a weak correlation between vitamin D and Ct.CaHigh R = 0.50, p = 0.02). Furthermore, no significant relationship between the dynamic histomorphometric bone formation indices (MS/BS, MAR, BFR/BS) could be observed (all correlations p > 0.05).

### OLS-Characteristics from Patients with axSpA

Significant differences in OLS-characteristics were observed when the total cohort was compared to reference data, as well as when both subgroups with and without mineralization defects were compared to reference. Outcomes and significant differences based on either t-test or Mann–Whitney rank sum test comparison or based on ANOVA or ANOVA on ranks are summarized in Suppl. Table 1, and Fig. 3.

The total cohort showed significantly higher OLS-area and OLS-perimeter in both cancellous and cortical bone compartments compared to reference. Additionally, OLS-density was lower in cancellous bone, and OLS-porosity and OLS-aspect ratio were elevated in cortical bone.

GROUP-1 revealed decreased OLS-density in the cancellous compartment, GROUP-2 had significantly higher OLS-porosity in cortical bone and lower OLS-density in cancellous bone. In both GROUP-1 and GROUP-2, OLS-area and OLS-perimeter were increased in both cancellous and cortical compartments compared to reference. In GROUP-1, additionally cortical OLS-aspect ratio was increased compared to reference. Furthermore, OLS-area of cortical bone was different between GROUP-1 and GROUP-2.

Similar to the BMDD findings, we could not detect any significant correlation of OLS-characteristics with disease-related parameters or serum phosphorus, vitamin D level or PTH (all p > 0.05).

For further information on the deviations from normal OLS-characteristics in GROUP-2, we compared OLS outcomes dependent on treatment (n = 9 received NSAID and n = 7 immunobiologic treatment) and found significant differences. Comparison of these two subgroups showed higher Cn.OLS-porosity (p = 0.024), higher Cn.OLS-area (p = 0.006), and higher Cn.OLS-perimeter (p = 0.002) in the subgroup with NSAID treatment. This subgroup comparison for Cn.OLS-area within GROUP-2 is shown in Fig. 4.

### Raman Microspectroscopy Outcomes

**Fig. 5:**
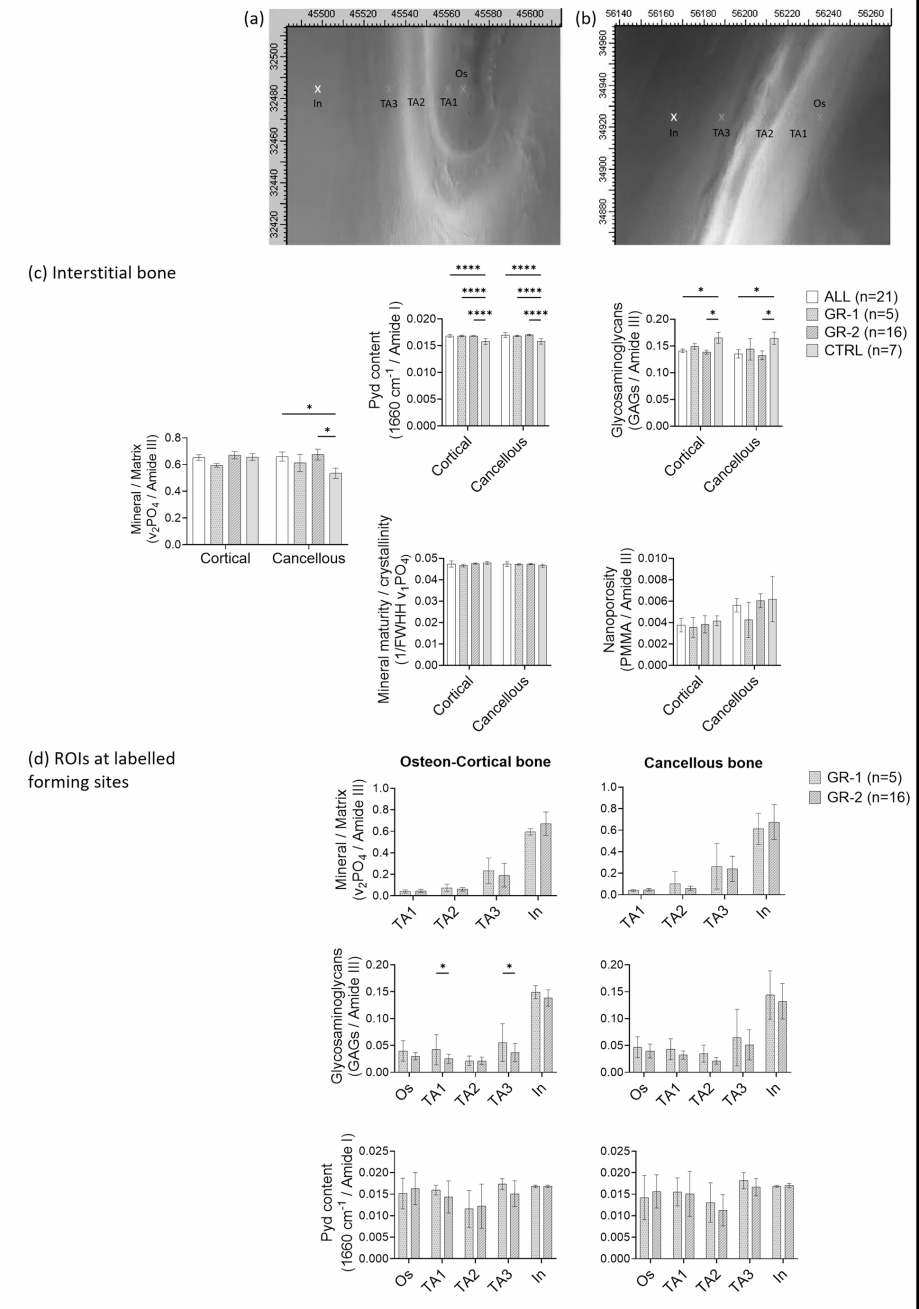
Raman microspectroscopy analysis. **a** Light microscope images (under UV light) showing the tetracycline labelled formation sites in cortical and **b** in cancellous bone from a patient of GROUP-2 (GR-2) demonstrating the ROIs selected for measurement. **c** Outcomes of selected areas of interstitial bone from patients in comparison to controls (CTRL). Differences in mineral:matrix (higher in cancellous bone, higher in the total cohort (ALL) versus CTRL, and higher in GROUP-2 versus CTRL), pyridinoline per organic matrix content (increased in cortical and cancellous bone, increased in ALL, GROUP-1, and GROUP-2 versus CTRL), and glycosaminoglycans per organic matrix content (decreased in cortical and cancellous bone, lower in ALL and GROUP-2 versus CTRL) were observed. **d** Outcomes of bone material properties at the defined ROIs: Os = Osteoid, TA1 = youngest mineralized matrix (formed after completion of second tetracycline label), TA2 = bone formed between administration of first and second label, TA3 = bone formed before the administration of first label, In = older bone matrix in a distance of 20–35 μm from TA3 (no defined tissue age). Data show mean ± SEM. P-value (*p < 0.05, ****p < 0.0001) indicates significant difference according to pairwise post-hoc tests following Two-Way ANOVA

Comparison of the Raman spectroscopic data in selected areas of older/interstitial bone from the total cohort versus control data (CTRL) revealed a significant (p = 0.0262) increase in the MM in the cancellous compartment. This increase was also observed separately in patients from GROUP-2 (those without mineralization defects) compared to the CTRL (p = 0.0166), as shown in Fig. 5c. No significant differences in this ratio were observed in the cortical bone between groups and subgroups (all p > 0.05). Pyridinoline (Pyd) enzymatic collagen cross-links were significantly higher in the total cohort compared to the CTRL group. This increase was also observed in patient subgroups (Fig. 5c), in both cortical and cancellous bone (all p < 0.0001). A reduction in GAG content was observed in GROUP-2 compared to the CTRL in both cortical (p = 0.0360) and cancellous bone (p = 0.0122), and the same was seen when the total cohort was compared with CTRL (p = 0.0487). MMC and nanoporosity were similar between the groups and subgroups across both bone compartments.

When analyzing actively bone forming regions between patient subgroups (Fig. 5d), no significant differences in the MM were observed between GROUP-1 and GROUP-2 in either osteons (p = 0.228) or cancellous bone (p = 0.605). As expected, MM values increased with tissue age. Compared to GROUP-2 (patients without mineralization defects), GROUP-1 (patients with mineralization defects) exhibited higher GAG content in both osteons and cancellous bone. Significant differences were observed in the osteon’s positions TA1 (the area between the second label and the mineralizing front) and TA3 (the area behind the first label), with elevated GAG levels between GROUP-1 (p = 0.046) and GROUP-2 (p = 0.029).

## Discussion

We have presented bone matrix mineralization and osteocyte lacunae as well as material / compositional characteristics in a biopsy cohort of male patients for further information about underlying skeletal abnormalities in axSpA. Ninety percent of our patients were positive for HLA-B27, a genetic condition, which is known to be highly associated with axSpA [1, 3]. Cortical bone volume by histomorphometry was decreased on average in our cohort, and about 50% of our patients presented with low BMD (by DXA) [12]. Given that in the previous report, five patients were identified by histology to have mineralization defects [12], we analyzed the subgroups with and without mineralization defects separately additionally to the consideration of the total cohort for comparisons. The patients with axSpA revealed lower bone matrix mineralization, showed indications of increased osteocyte lacunae size and had higher pyridinoline content of the bone matrix. When considering the subgroups with and without mineralization defects separately, the differences in osteocyte lacunae and pyridinoline content compared to healthy individuals were visible in both subgroups, while entire bone matrix mineralization was altered only in the subgroup with histologically defined mineralization defects.

The bone matrix mineralization, as obtained by the bone mineralization density distribution (BMDD) in the present work, is a characteristic that is essentially dependent on the bone turnover/formation as well as on the mineralization processes (i.e. the speed and final level of mineral accumulation within each newly formed bone packet) [17]. Relatively higher bone formation (and consequently lower average tissue age) and/or decelerated bone mineralization shift the BMDD to lower calcium concentrations and vice versa. In the current work, we found a strong correlation between the cancellous and cortical BMDD from each patient, suggesting a tight coupling of the aforementioned processes in both bone compartments of each individual. This is interesting to note, as reduced mass/volume was in particular observed in the cortex from patients with axSpA suggesting increased resorption in this compartment [12, 21].

We found that GROUP-1 (subgroup with histologically defined mineralization defects) presented with abnormally low bone matrix mineralization. These five patients had in particular a highly increased percentage of lowly mineralized bone matrix and a generally large variation in bone mineralization heterogeneity. At first glance, this might appear not surprising as the histologic evaluation of these samples already indicated a mineralization defect. However, the histologic/histomorphometric analysis of mineralization defects is based on the evaluation of the osteoid (i.e. abnormal amount and thickness of osteoid, together with elevated mineralization lag time of the matrix), thus does not provide any information about the degree and distribution of mineral in the mineralized matrix. We have now found evidence for inadequate degree and distribution of calcium within the mineralized bone matrix of these patients. As an aside, osteoidosis and abnormally low bone matrix mineralization are not mutually dependent, as shown in a patient group with a rare bone disorder [22]. Given that our five patients with mineralization defects did not reveal any signs of increased bone turnover/formation by histomorphometry, their lower bone matrix mineralization cannot be attributed to lower bone tissue age but indicates alteration in their mineralization processes. In contrast, the second group of the axSpA patients GROUP-2 (those without mineralization defects), had on average normal bone mineralization suggesting both normal bone turnover/formation and mineralization processes. Their histomorphometric outcomes of dynamic bone formation, in particular mineralizing surface which is a primary index for bone formation, suggests lower or normal bone formation depending on the reference cohort used for comparison [12, 23].

It is also interesting that no significant correlation of the BMDD outcomes with histomorphometric indices of bone formation (MS/BS, MAR, BFR/BS) could be observed, although it is known (as mentioned before) that bone matrix mineralization is dependent on tissue age. The reason of the lack of this (otherwise usually observed [17]) correlation remains unknown, however might be related to a relatively large variation of mineralization kinetics (i.e. the speed of mineral accumulation in the organic matrix) in this patient group.

Raman-determined material/compositional data obtained in the present work give some complementary information about the bone material maturation additionally to the BMDD data obtained from the entire cross-sectional bone area. The Raman data showed no essential differences in the maturation of young or older bone tissue between the patients’ subgroups (with versus without mineralization defects). The comparison of the material properties in selected older bone tissue of the patients with those from healthy individuals, however, indicated some differences. Specifically, the MM ratios were significantly higher in the total cohort and in GROUP-2 compared to controls in interstitial cancellous bone. Noteworthy, the Raman microspectroscopy measure of mineral is the phosphate peak, which is not only sensitive to the amount of mineral but also to the chemical maturation of the mineral phase in bone. These MM differences seem to be confined to specific older tissue ages (and in turn to limited bone area) as no BMDD abnormalities were observed for GROUP-2. Additionally, we want to mention that in a previous work about the transgenic HLA-B27 rat model [11], we observed lower MM ratios at bone forming sites (adjacent and between the fluorescent labels). Due to our study design of the present work, we have no information how the MM ratios of the patients compare with those from the controls at bone forming sites. Furthermore, there might be general bone material related differences between the animal model and the human disorder, as the animal model is lacking any histologic signs of mineralization defects such as increased osteoid volume [24].

Another observations at selected older bone areas in our patients include the reduced amount of GAGs. Lower GAG content was linked to deterioration of bone toughness (associated with reduced tissue water content) [25]. Moreover, a novel finding was the increased level of Pyd in patients with axSpA compared to the CTRL. Pyd is a mature collagen cross-link that stabilizes the collagen fibers, yet increased enzymatic collagen cross-links associate with collagen fibers exhibiting brittle behavior at high mechanical strains [26]. This elevated Pyd level is in agreement with the previous observations at bone forming sites in the rat model of ankylosing spondylitis [11], however, the mechanism for this alterations remains to be elucidated. Noteworthy, about 90% of our patients were positive for the HLA-B27 gene, which is a risk factor for a group of chronic inflammatory autoimmune diseases including inflammatory bowel disease. There is evidence that HLA-B27 may alter the gut microbiome [27, 28] e.g. which in turn also may affect bone properties [29], which, however, remains speculative in our patients as the observed deviations from normal are from small interstitial bone areas.

Apart from the information about bone material, we measured osteocyte lacunae characteristics in the bone tissue and found highly significant alterations of OLS characteristics, in particular increased OLS size in the total cohort as well as in both patient groups with axSpA. Larger OLS-area was previously also observed in osteomalacic bone in XLH [30]. Mineralization deficits in particular in perilacunar bone resulted in an increased area of the OLS, as measured by qBEI [30]. Consequently, we might hypothesize that the increased OLS-area found in GROUP-1 (those with mineralization defects) generally reflects the defect in bone matrix mineralization comparable to the (albeit much more severe) observations in XLH [30]. However, in GROUP-2 without mineralization defects, the reason for the altered OLS size in a bone matrix with normal bone mineralization is unclear. In a previous work reporting on bone in chronic kidney disease, we found larger OLS-area in those patients with increased parathyroid hormone levels and histomorphometric high turnover [31], suggesting that in situations when high calcium concentrations are required, i.e. high bone formation in the latter case, the osteocyte might release calcium from its surrounding matrix, termed osteocytic osteolysis. Therefore, we also analyzed correlations between the OLS-characteristics and PTH in our patients, however, did not find a significant relationship. In this context, it has to be mentioned, that albeit the parathyroid levels were increased in some of our patients with axSpA, the range of PTH levels was smaller than in the aforementioned other patient group. While the maximum PTH level in the present cohort with asSpA was 119.4 pg/dL, extremely increased levels (up to tenfold of the current) were found in the previously studied patients with kidney disease [31].

Principally, the morphologic alterations of osteocyte lacunae might be also indicative for a role of osteocyte dysfunction in axSpA. Indeed, alterations in osteocyte function and altered protein expression have been observed [32]. Sclerostin in serum as well as in bone tissue from joints analyzed by immunohistochemistry was lower in patients with ankylosing spondylitis. In particular, the tissue analysis gave evidence for a strikingly low percentage of sclerostin-positive osteocytes in this disorder compared to controls. While in control samples, about 30% of the osteocytes were negative for sclerostin, in patients with ankylosing spondylitis, these were nearly 80% of the osteocytes without any increase in percentage of empty lacunae [32]. We do not have direct evidence that changes in sclerostin, or other protein expression might directly be related to larger lacunae size, but based on observations in other disorders [33] we speculate that altered lacunae morphology might be indicative for an altered function of osteocytes.

When considering the subgroup with normal BMDD, it is noteworthy, that the patients under immunobiologic treatment had smaller OLS size than those with NSAID treatment. We are aware that the number of patients is rather small for this analysis, however, we report the finding here as it might indicate that the osteocyte function/morphologic abnormalities are changed towards normal under immunobiologic treatment. Furthermore, in the previous histomorphometric findings from our current patient cohort [12], a link to phosphorus metabolism was reported, which was not observed for bone matrix mineralization or osteocyte lacunae morphology.

Several questions remain in this study. Most important, it is unclear why part of the patients with axSpA had developed mineralization defects while others did not. Cases of patients with axSpA and osteomalacia are also reported in the literature [13, 14, 34–36]. Actually, small signs of local mineralization defects (for instance relatively low mineralized cement lines or small areas with low mineralization within otherwise accordingly mineralized bone packets) were seen also in the qBEI images from patients of GROUP-2. These features did not significantly influence the BMDD yet, however might do so during progression of the disease. In any case, the patients of GROUP-1 did not present with any obvious clinical abnormalities compared to the remaining cohort as discussed in the previous publication [12]. In particular, the patients with mineralization defects did not present with vitamin D deficiency. The latter is important to note, as vitamin D deficiency associated with osteoidosis was found, for instance, in about 25% of the general population in Germany [37], which is in frequency approximately the same as in the present cohort with axSpA.

Our study has several limitations, including its restriction to male patients, which is owing to the fact that radiographic axSpA is a male predominant disorder and osteoporosis in axSpA is more prevalent in men [38]. Bone tissue/material properties from female patients with axSpA are of similar interest, however, potential interference of menopause with disorder effects would have to be considered. In this context, we have to mention that the BMDD- and OLS-reference cohorts included also women or contained exclusively women, respectively. While for the BMDD outcomes no gender-differences in healthy subjects have been observed as reported previously [39], it is unknown whether OLS-characteristics differ between healthy women and men. Unfortunately, OLS-characteristics from healthy male subjects have not been studied yet systematically, and the only OLS reference data available so far are those used in the current work. Generally, one age- and gender-matched control biopsy group for comparison with the patients would be advantageous for biopsy outcomes, for which no established reference data exist. Another important limitation is the lack of specific serum data, which would have been of interest in relation to the presented material data. In particular, sclerostin serum levels would have been of interest for more information about bone turnover and progression of structural changes in axSpA [32, 40] but unfortunately could not be evaluated in this cross-sectional study. Furthermore, the number of bone biopsy samples studied was rather low, albeit it was higher compared to previous biopsy studies in axSpA. The latter together with the heterogeneity of findings (considering the presence of mineralization defects in a subgroup of patients) makes subgroup-comparisons and definite conclusions on alterations of bone tissue and material characteristics in axSpA in general as well as the link to bone fragility in this disorder problematic.

Noteworthy, our patients were not treated with bisphosphonates (as this was an exclusion criteria for the participants of this study). It is further unclear how this treatment, which is frequently considered for ankylosing spondylitis, will affect the bone properties. Bisphosphonates are currently prescribed not only in order to treat osteoporosis in these patients, they also have immunomodulatory effects, which might have potential beneficial effects against the inflammation in this disorder, albeit the results of clinical studies are not definite [41, 42]. According to the current work and data from the literature [2, 12–14], patients with axSpA commonly do not present with increased bone turnover, thus, it is unclear whether bisphosphonate treatment would significantly alter their bone tissue/material properties. For instance, an increase in bone mineral content (potentially exceeding normal levels) might be expected after bisphosphonate treatment [43]. Most important, it is unclear whether bisphosphonate use might have any beneficial effect in patients with mineralization defects/osteomalacia. 

In conclusion, in the current work we have observed two groups of patients with axSpA. The group with osteoid-related histomorphometric evidence of mineralization defects showed abnormally low degree and increased heterogeneity of bone matrix mineralization. The other group without evidence of mineralization defects presented with normal bone matrix mineralization. However, the Raman data showed that in axSpA, even without visible mineralization defects, there may be early changes in the bone matrix, such as increased levels of Pyd, and reduced GAGs, that may lead to poor bone quality and further contribute to bone fragility in axSpA. Strikingly, both patient groups with and without mineralization defects had abnormal osteocyte lacunae characteristics, including larger size of the osteocyte lacunae in both cancellous and cortical compartments. These morphological deviations from normal might be indicative that in axSpa, the bone cell with its function compromised, is the osteocyte. Therefore, our data hypothesize an osteocyte signature, characterized by low bone turnover and mineralization impairment with increased lacunae size and altered organic mineral matrix, in men with axial SpA. However, our data cannot be validated in women with axSpA, especially in post-menopausal women, and new studies need to be carried out to explore the differences between sexes for a further understanding of skeletal alterations in axSpA, which is definitely required for evaluation of treatment options.

## Supplementary Information

Below is the link to the electronic supplementary material.

Supplementary Material 1.

## Data Availability

The research data supporting this publication can be accessed at our institutional digital data repository for published research via https://creed.lbg.ac.at.
