# Supplementary material for: Increased Osteocyte Lacunae Size and Organic Matrix Pyridinoline Content in Transiliac Bone from Patients with Axial Spondyloarthritis (axSpA)
Source: Calcif Tissue Int. 2025 Dec 1;116(1):138. doi: 10.1007/s00223-025-01446-x (PMC12669350; doi:10.1007/s00223-025-01446-x)
Supplement: Supplementary file 1 — Supplementary Material 1. [file 223_2025_1446_MOESM1_ESM.docx]

*Suppl. Table 1:* Cancellous and cortical BMDD and OLS outcomes from patients with axSpA compared to reference

|  |  | axSpA  total cohort  (n=21) | axSpA with min defect^1^  (GROUP-1, n=5) | axSpA without min defect  (GROUP-2, n=16) | adult REF^2^ | ANOVA or  ANOVA on ranks  p-value |
| --- | --- | --- | --- | --- | --- | --- |
| Cancellous | CaMean (wt%) | 22.88 ^#^  [22.37; 23.29] | 22.11 **,°  [20.628; 22.52] | 23.19  [22.64; 23.38] | 23.31  [23.05; 23.70] | 0.004 |
|  | CaPeak (wt%) | 23.74  [23.48; 24.26] | 23.05 **,°  [21.92; 23.66] | 24.00  [23.61; 24.39] | 24.09  [23.83; 24.44] | 0.008 |
|  | CaWidth (Δwt%) | 3.99 ^#^  [3.81; 4.42] | 4.85 ***,°  [4.42; 5.63] | 3.99  [3.81; 4.12] | 3.81  [3.73; 3.99] | 0.001 |
|  | CaLow (%B.Ar) | 6.26 ^##^  [4.67; 7.58] | 7.73 **  [7.22; 18.72] | 5.25  [4.56; 6.85] | 4.61  [3.95; 5.57] | 0.002 |
|  | CaHigh (%B.Ar) | 2.98  [1.76; 4.76] | 1.75 *  [0.76; 2.84] | 3.83  [2.56; 5.05] | 3.99  [2.76; 6.08] | 0.032 |
|  | OLS-porosity (%) | 0.474  [0.426; 0.501] | 0.438  [0.362; 0.532] | 0.481  [0.434; 0.501] | 0.450  [0.412; 0.514] | ns |
|  | OLS-density (Nb.mm^-2^) | 167.5 ^###^  (25.0) | 153.5 **  (44.6) | 171.8 *  (14.7) | 189.2  (23.1) | 0.001 |
|  | OLS-area (μm^2^) | 23.01 ^###^  (2.4) | 23.91 **  (2.42) | 22.73 **  (2.45) | 20.90  (1.87) | <0.001 |
|  | OLS-perimeter (μm) | 21.79 ^###^  [20.70; 22.55] | 22.64 ***  (1.66) | 21.46 **  (1.15) | 20.42  (0.89) | <0.001 |
|  | OLS-aspect ratio | 2.49  [2.41; 2.61] | 2.47  [2.34; 2.58] | 2.50  [2.45; 2.61] | 2.54  [2.40; 2.62] | ns |
| Cortical | CaMean (wt%) | 23.01  [22.55; 23.27] | 22.08 **,°°  (0.76) | 23.09  (0.38) | 22.96  (0.57) | 0.002 |
|  | CaPeak (wt%) | 24.00  [23.53; 24.18] | 23.22 *,°  (0.69) | 24.01  (0.34) | 23.89  (0.54) | 0.012 |
|  | CaWidth (Δwt%) | 3.99  [3.86; 4.42] | 4.59 *,°°  [4.42; 5.98] | 3.99  [3.81; 4.12] | 4.07  [3.81; 4.25] | 0.003 |
|  | CaLow (%B.Ar) | 5.86  [4.42; 7.02] | 7.81 *,°  [6.74; 13.74] | 4.88  [4,33; 6,47] | 5.19  [4.71; 6.51] | 0.012 |
|  | CaHigh (%B.Ar) | 3.74  [2.16; 4.59] | 1.66  [1.04; 3.85] | 3.88  [2.87; 4.90] | 3.70  [2.12; 4.80] | ns |
|  | OLS- porosity (%) | 0.519 ^##^  (0.073) | 0.498  (0.102) | 0.526 *  (0.064) | 0.460  (0.085) | 0.020 |
|  | OLS-density (Nb.mm^-2^) | 188.0  (37.6) | 159.7  (32.7) | 196.8  (35.4) | 208.8  (46.7) | 0.053 (ns) |
|  | OLS-area (μm^2^) | 23.29 ^###^  [21.34; 26.78] | 26.70 ***,°  (3.83) | 23.09 ***  (2.94) | 18.48  (2.51) | <0.001 |
|  | OLS-perimeter (μm) | 21.79 ^###^  [19.64; 23.91] | 23.40 ***  (2.37) | 21.26 ***  (2.09) | 18.98  (1.61) | <0.001 |
|  | OLS-aspect ratio | 2.37 ^#^  (0.24) | 2.52 (0.19)* | 2.32  (0.24) | 2.25  (0.20) | 0.019 |

Data shown mean (SD) or median [25^th^; 75^th^ percentiles] for normally or non-normally distributed cohort data, respectively.

^1^ Definition of mineralization defect: O.Th > 12.5μm and Mlt > 100days

^2^ Adult reference data published previously: OLS-REF, n=52 [18]; BMDD-REF, n=25 [16]

*p<0.05, **p<0.01, ***p<0.001 versus REF, and °p<0.05, °°p<0.01 versus GROUP-2 based on post-hoc pairwise comparison following ANOVA or ANOVA on ranks

^#^p<0.05, ^##^p<0.01, ^###^p<0.001 based on t-test or Mann-Whitney rank sum test for comparison between the total cohort and the corresponding reference.
